# Governing relational opportunity in hybrid work: structural relational deprivation and the reproduction of workplace inequality

**DOI:** 10.3389/fsoc.2026.1955187

**Published:** 2026-09-30

**Authors:** Nthabeleng Innocentia Mdhluli

**Affiliations:** Department of Industrial and Organisational Psychology, University of South Africa, Pretoria, South Africa

**Keywords:** employee relations, employee voice, hybrid work governance, relational inequality, relational opportunity, structural relational deprivation

## Abstract

Hybrid work changes not only where employees work but also how they access the relationships through which information, support, development and recognition are obtained. This article argues that employee relations scholarship has paid insufficient attention to how organisations govern and distribute this access. It introduces relational opportunity governance, defined as the formal and informal rules, managerial decisions, technological arrangements, resource allocations and voice institutions through which access to employment-relevant relationships is enabled, constrained and contested. It also develops structural relational deprivation, a work-system condition in which such access is persistently, systematically and consequentially restricted or unevenly distributed across identifiable groups, roles or organisational arrangements. Drawing on a critical integrative review of South African and international scholarship on hybrid work, employee relations, organisational inequality, workplace networks and digital communication, the article identifies eligibility, temporal coordination, developmental and informational arrangements, performance-evaluation and recognition arrangements, material provision and voice institutions. It proposes that restricted relational access may weaken employees’ network position, relational confidence, visibility and organisational influence, thereby reducing their capacity to exercise effective voice and challenge the arrangements that produced the disadvantage. Exposure may vary across occupational, organisational and materially differentiated groups, although demographic patterns require direct empirical investigation. The article reconceptualises hybrid work as a question of governance, power and distributive fairness rather than location or communication frequency. Six propositions and a provisional governance typology are advanced to guide future multilevel and longitudinal research.

## 1 Introduction

Hybrid work has become an established feature of employment in professional and knowledge-intensive occupations, offering employees greater control over how they organise work and non-work responsibilities, and offering organisations access to wider labour markets and greater operational continuity. A randomised trial involving 1,612 employees found that a coordinated two-day work-from-home arrangement improved job satisfaction and reduced turnover without compromising performance (Bloom et al., 2024), and work-from-anywhere arrangements have generated productivity gains where job requirements, technology and management practices were aligned (Choudhury et al., 2021). Meta-analytic evidence associates telecommuting with greater autonomy and lower work–family conflict, although relational difficulties become more likely as remote-work intensity increases (Gajendran and Harrison, 2007). These findings show that hybrid work can produce meaningful benefits, but that such benefits depend on how the arrangement is designed, supported and implemented.

The significance of hybrid work extends beyond flexibility and productivity. By changing where and when work is performed, hybrid arrangements reshape employees’ access to relationships that carry substantive employment value: informal knowledge, practical support, mentoring, sponsorship, recognition, career visibility and participation in organisational decision-making. These forms of access are not preserved automatically when work becomes more dispersed. Organisation-wide remote work has been associated with collaboration networks that become more static and fragmented across organisational silos (Yang et al., 2022); professional isolation has been linked to poorer performance and stronger turnover intentions as telework intensity increases (Golden et al., 2008); and research among hybrid workers indicates that informal communication and social support matter more for relational outcomes than the overall volume of organisational communication (Mäkelä et al., 2024). Taken together, this evidence does not show that hybrid work is inherently detrimental to workplace relationships. It shows that employees may remain formally included in organisational communication while having unequal access to the relationships through which information, support, development and influence circulate.

The central employee relations question is therefore not whether hybrid work is beneficial or harmful, but how organisations govern the relational opportunities associated with it and how the consequences of that governance are distributed. Eligibility rules determine who may work remotely and on what terms. Scheduling arrangements shape whether employees have predictable access to colleagues, supervisors and decision-makers. Managerial discretion shapes whose flexibility is accommodated and whose contribution remains visible. Performance systems determine whether mentoring and knowledge-sharing are recognised as legitimate contributions. Material resources determine whether employees can participate consistently in digitally mediated work, and voice arrangements determine whether employees can question or challenge the conditions under which hybrid work is implemented.

South African research illustrates why these questions cannot be reduced to individual preference or communication style. Employees report greater flexibility, engagement and well-being where hybrid arrangements are supported by clear policies and consistent organisational support (Bartlett et al., 2026; Charles et al., 2025; Kokt and Seqhobane, 2024), while other studies point to isolation, extended working hours, policy misalignment and unequal access to reliable digital infrastructure (Botha and Coetzee, 2022; Ramantswana, 2025; Saurombe et al., 2022; Winkler-Titus et al., 2025). These contrasting findings suggest that the outcomes of hybrid work depend less on work location than on the wider organisational and material conditions under which flexibility is governed and experienced. The South African context sharpens this concern further: hybrid work is being introduced into a labour market already marked by substantial occupational, socioeconomic and demographic inequality, so that a single formal policy is likely to produce very different levels of access depending on employees’ infrastructure, prior relational capital, organisational status and proximity to influential networks. Formal equality in policy may consequently coexist with substantive inequality in practice.

These conditions position hybrid work within the field of employee relations because decisions about eligibility, temporal coordination, developmental and informational arrangements, performance-evaluation and recognition arrangements, material provision and voice institutions engage questions of managerial authority, employee voice and distributive fairness. A hybrid arrangement may appear generous because it offers flexibility while still disadvantaging employees excluded from mentoring, informal knowledge flows, sponsorship and decision-relevant networks. The relevant question is therefore not only whether employees may work flexibly, but whether they retain fair and usable access to the relationships through which employment opportunities are realised.

Existing literature does not yet provide an adequate account of this problem. Hybrid-work research has focused mainly on the frequency or location of remote work, well-being, performance and managerial support, offering only a partial explanation of how organisational rules govern relational access within the employment relationship. Workplace loneliness research explains employees’ subjective experience of relational deficiency but does not establish whether the underlying opportunity structure is accessible, fair or unevenly distributed. The unresolved problem concerns the work-system arrangements through which relational opportunities are governed, allocated and reproduced. Previous work by the present author has examined how digitally mediated interaction can contribute to organisational loneliness when frequent communication fails to provide meaningful connection and belonging (Mdhluli, 2026). That article addresses an individual-level psychological experience arising from the character of digital interaction. The present article addresses a different theoretical problem at a different level of analysis: how hybrid-work arrangements govern access to relationships that carry information, development, recognition, influence and voice. Workplace loneliness is treated here as one possible individual-level consequence of unequal relational access rather than as the defining phenomenon.

### 1.1 Aim, motivation and objectives

This article develops an employee relations perspective on hybrid work as a contested and distributive arrangement within the employment relationship. It draws on the pluralist tradition, which recognises that employers and employees have overlapping but not identical interests, and that managerial authority, although legitimate, is open to contestation where it affects working conditions, access to organisational resources and employee voice (Ackers, 2014; Kaufman, 2020). From this perspective, flexibility is not simply a benefit offered by management; it forms part of the substantive terms of employment and may become an object of consultation, negotiation, regulation and dispute.

The article introduces relational opportunity governance to describe the rules, managerial decisions, work practices and voice arrangements through which employees’ access to employment-relevant relationships is organised, directing attention to who controls these opportunities, how they are allocated, and whether employees can influence the arrangements that shape their access. Within this framework, structural relational deprivation refers to a work-system condition in which organisational arrangements systematically restrict or unevenly distribute employees’ access to employment-relevant relationships. The construct does not describe an employee’s subjective feelings, communication frequency or network size; it identifies a structural shortfall in the opportunities and resources from which workplace relationships can be formed, maintained and used, and it may be evident across an organisation or concentrated within particular occupational, structural or demographic groups.

The construct is needed because neither the availability of hybrid work nor the volume of organisational communication reveals whether relational opportunities are distributed fairly. Employees may enjoy considerable autonomy while remaining excluded from mentoring and influential networks; others may interact frequently with colleagues while having little opportunity to shape decisions about their own working arrangements; and whether employees ineligible for hybrid work carry additional coordination burdens without receiving its benefits is a further, currently untested distributive question this article treats as a direction for future research (Section 5.3) rather than an established finding. These are questions of control, allocation, implementation, voice and fairness within the employment relationship, not simply of individual preference or managerial technique.

The article pursues three objectives: to define structural relational deprivation and distinguish it from adjacent constructs, including social capital, network disadvantage, relational coordination, workplace loneliness, professional isolation, workplace exclusion, low organisational belonging and relative deprivation; to develop a framework explaining how eligibility, temporal coordination, developmental and informational arrangements, performance-evaluation and recognition arrangements, material provision and voice institutions govern relational opportunity in hybrid work; and to explain how unequal access may become institutionally reproduced when reduced realised visibility to decision-makers and organisational influence limit employees’ capacity to contest or reshape disadvantageous arrangements. Correspondingly, it makes three contributions: a system-level construct for examining unequal access to employment-relevant relationships; relational opportunity governance as a framework for analysing hybrid work in relation to managerial prerogative, employee voice and distributive fairness; and an institutional reproduction process explaining how unequal relational access can become self-sustaining. In the South African context, this framework provides a basis for examining how hybrid work intersects with occupational stratification, infrastructural inequality and historically patterned access to organisational power.

## 2 Literature review and theoretical foundation

### 2.1 Employment-relevant relationships and relational opportunity

Workplace relationships are not only sources of companionship and belonging; they provide access to resources with direct employment value, including informal knowledge, practical assistance, feedback, mentoring, sponsorship, recognition, career visibility and influence over organisational decisions. Social-capital scholarship has long shown that resources embedded in workplace networks enhance knowledge exchange, career mobility and access to organisational opportunities (Nahapiet and Ghoshal, 1998; Seibert et al., 2001), and Burt’s (2004) work on structural holes shows that employees who connect otherwise separated parts of an organisation gain informational and reputational advantages unavailable through formal job structures alone.

These relational resources remain important in hybrid work, but the conditions of access have changed. Lauring and Jonasson (2025) argue that hybrid work cannot be reduced to physical location because it involves movement across spatial, temporal and communicative dimensions simultaneously, a distinction that matters because employees with formally identical hybrid arrangements may experience very different relational access depending on how these dimensions combine. Wu et al. (2023) show that advice-seeking relationships were less likely to be maintained where advice seekers and providers differed in their perceptions of communication-technology usability, an effect that was stronger for advice seekers who worked remotely more frequently, suggesting that apparently uniform technological arrangements can produce unequal relational consequences. Begemann et al. (2024) similarly find that remote informal communication depends on an interdependent combination of technological, social and individual conditions, cautioning against assuming that digital tools automatically reproduce the spontaneous interaction that occurs more readily in shared workplaces.

Taken together, these studies suggest that employees’ relational position cannot be inferred from attendance patterns or communication frequency alone; what matters is whether they have practical and sustainable access to relationships through which organisationally valuable resources circulate. This article uses relational opportunity to refer to employees’ usable access to workplace relationships that provide information, support, development, recognition, influence and voice. The concept is related to social capital but not interchangeable with it: social capital concerns the resources embedded in relationships and networks, whereas relational opportunity concerns the organisational conditions that enable employees to form, maintain and use those relationships. An organisation may possess substantial collective social capital while particular employees remain unable to enter the networks in which it circulates. The theoretical question is therefore not only whether relational resources exist, but how access to them is organised and distributed.

### 2.2 Relational opportunity governance

This article defines relational opportunity governance as the formal and informal rules, managerial decisions, technological arrangements, resource allocations and employee-voice mechanisms through which access to employment-relevant relationships is enabled, constrained and contested. The concept moves the analysis beyond the number of days employees work remotely: two employees may spend the same proportion of their week away from the office yet experience markedly different relational conditions because their schedules, job status, managerial arrangements, technological access and opportunities for voice differ. Relational opportunity is consequently shaped by a configuration of governance arrangements rather than by hybrid-work intensity alone, and six interdependent dimensions of this configuration can be distinguished. These dimensions are eligibility governance, temporal governance, developmental and informational governance, performance-evaluation and recognition governance, material governance and voice governance.

*Eligibility governance* concerns decisions about which jobs and employees qualify for hybrid work and whether approval depends on managerial discretion. Lauring and Jonasson’s (2025) distinction between individual work-mode choices and collective patterns of hybrid work matters here, because an employee’s formal flexibility may operate within a team where access is highly asymmetrical, so that a policy can appear neutral at the individual level while reproducing occupational or hierarchical differences at the collective level.

*Temporal governance* concerns the organisation of shared working time. Relational access depends not on whether employees attend a central workplace, but on whether relevant colleagues and decision-makers are available at the same time. Individually chosen office days may preserve autonomy while producing little meaningful co-presence, whereas uncoordinated remote schedules may reduce access to mentoring even where formal meetings continue. Bloom et al. (2024) randomised trial shows that a coordinated arrangement can improve retention and satisfaction without damaging performance, supporting the view that outcomes depend on structured implementation rather than remote work per se.

*Developmental and informational governance* concerns access to mentoring, sponsorship, informal advice and cross-functional networks. Keppler and Leonardi (2023) show that employees require relational confidence before they can convert awareness of expertise into successful knowledge-seeking, and Wu et al. (2023) show that advice ties weaken where employees differ in their ability to use communication technologies; together these findings indicate that formal digital access does not guarantee equal access to organisational knowledge. Structured onboarding can reduce reliance on informal proximity: Tutar et al. (2026) found that social-media-supported onboarding and microlearning were associated with role clarity, belonging and performance in hybrid work, with psychological safety shaping their effectiveness. These relational resources should be distinguished from formal development outcomes, such as selection for training, stretch assignments or promotion, which may follow from employees’ access to, or exclusion from, developmental relationships.

*Performance-evaluation and recognition governance concerns* the formal and informal arrangements through which employees’ contributions are documented, interpreted and considered in decisions concerning feedback, recognition, training and advancement. These arrangements include the performance criteria applied, the sources of evidence available to managers, the consistency with which standards are implemented across work locations and the extent to which relational contributions, such as mentoring and knowledge-sharing, are recognised as legitimate organisational work. This governance dimension is analytically distinct from employees’ realised visibility to decision-makers. Performance-evaluation and recognition arrangements are organisational antecedents that shape whether employees’ contributions become visible and credible, whereas realised visibility is an intermediate relational condition through which those arrangements may influence later employment outcomes. Kasperska et al. (2024) demonstrate the importance of this distinction. In their experimental study of more than 1,000 managers, employees described as working from home were less likely than otherwise comparable office-based employees to be selected for promotion, salary increases and training. These disadvantages were smaller where working from home was common within the team, indicating that managerial interpretation and organisational norms influence how employee contributions are evaluated. The findings do not establish that remote work inevitably reduces visibility; rather, they show that evaluation and recognition arrangements may convert differences in work location into differences in organisational opportunity. South African evidence similarly emphasises the importance of evaluation practices rather than physical observation. Mabaso and Manuel (2024) found that remote and hybrid performance management required clear expectations, continuous communication and consistent standards across work locations. Wiatr (2026) further shows that managerial presence can be sustained through responsive engagement, relationship-building and practical support without continuous physical co-location. Taken together, these studies indicate that equitable hybrid-work governance requires evaluation systems that make contribution assessable without treating physical presence, immediate online responsiveness or informal managerial familiarity as proxies for performance.

*Material governance* concerns the infrastructure required for meaningful participation: reliable electricity, connectivity and workable physical space. Begemann et al. (2024) identify technological accessibility as an enabling condition for informal remote communication, while Ramantswana (2025) shows that South African teleworking is shaped by electricity disruptions and inconsistent organisational support, conditions that affect not only task completion but whether employees remain visible and can participate in developmental exchanges.

*Voice governance* concerns employees’ capacity to influence, question or challenge the arrangements that shape relational access, through both direct mechanisms and collective mechanisms involving trade unions and representative structures (Wilkinson et al., 2020). O’Brady et al. (2025) provide important comparative evidence that collective worker voice shaped whether remote performance-management systems in digitalised service work produced mutual gains or intensified managerial control, showing that technology alone did not determine the employment outcome. South African evidence adds an interpersonal dimension: Mohase et al. (2025) found that psychological safety mediated the relationship between inclusive leadership and employee voice among remote and hybrid employees, while Ngubane and Mbokota (2025) and Magwentshu et al. (2026) show that leaders’ emotional intelligence, accountability, trust and perceived interpersonal risk shape whether employees are willing to use available voice channels at all.

These six dimensions are analytically distinguishable but operate as an interdependent governance configuration. Coordinated schedules may produce little relational benefit where employees remain excluded from mentoring, sponsorship or influential networks. Developmental programmes may be ineffective where employees lack the infrastructure or psychological safety required to participate, while a formally inclusive hybrid-work policy may continue to produce unequal access where managerial discretion is extensive or performance-evaluation and recognition arrangements remain inconsistent or proximity-dependent. Relational opportunity governance must therefore be examined as a configuration of mutually reinforcing organisational conditions rather than as a checklist of isolated practices.

### 2.3 Structural relational deprivation: a sociological construct

The construct developed here is derived from, rather than added to, sociological theorising on organisational inequality. Acker’s (2006) concept of inequality regimes holds that organisations sustain unequal outcomes through interrelated practices and processes, many of which appear routine or formally neutral rather than deliberately discriminatory; eligibility rules, scheduling norms and informal sponsorship are precisely the kind of “neutral” organisational practice through which Acker argues inequality is produced and legitimated. Relational inequality theory extends this by specifying the mechanism: organisational resources, rewards and respect are distributed through relational processes, including social closure, in which incumbents restrict access to valued positions and networks, and claims-making, in which actors with greater organisational power are better positioned to have their claims on resources recognised (Tomaskovic-Devey and Avent-Holt, 2019). Applied to hybrid work, these two sociological accounts jointly imply that access to employment-relevant relationships, the informal knowledge, mentoring, sponsorship and decision-relevant networks discussed in Section 2.1, is itself a valued organisational resource whose distribution is governed, contested and capable of being closed off to particular groups through processes that need involve no deliberate exclusion at all. Structural relational deprivation is the name given to the patterned outcome of this governance and closure process specifically as it operates on relational access, and the institutional reproduction mechanism developed in Section 2.6 is the specific form that claims-making and social closure take once relational access has already been restricted.

Structural relational deprivation is accordingly defined as a work-system condition in which the governance of work systematically restricts or unevenly distributes employees’ access to relationships that provide information, support, development and recognition. In this definition, development refers specifically to access to mentoring, sponsorship, developmental advice, feedback and informal learning relationships. It does not include formal organisational decisions concerning training allocation, stretch assignments, promotion or career progression, which are treated as downstream employment outcomes. The construct refers to the work arrangement rather than to an individual employee’s psychological state; its organisational referent comprises the policies, managerial practices, scheduling patterns, technological systems, performance processes and material conditions through which relational access is organised, and it may therefore be observed across an organisation, team, occupational category or role configuration even though its consequences may be concentrated among particular groups. Consistent with relational inequality theory’s emphasis on social closure operating through ordinary practice, the definition deliberately excludes influence and voice from its content: influence and voice are treated instead as downstream mediating conditions, shaped by deprivation and in turn shaping whether deprivation can be contested, rather than as constitutive elements of the construct itself. This separation is necessary to avoid circularity between the construct and the outcomes it is proposed to explain, and Section 2.6 and Figure 1 make the resulting four-layer structure, governance antecedents, structural relational deprivation, mediators and outcomes, explicit.

**Figure 1:**
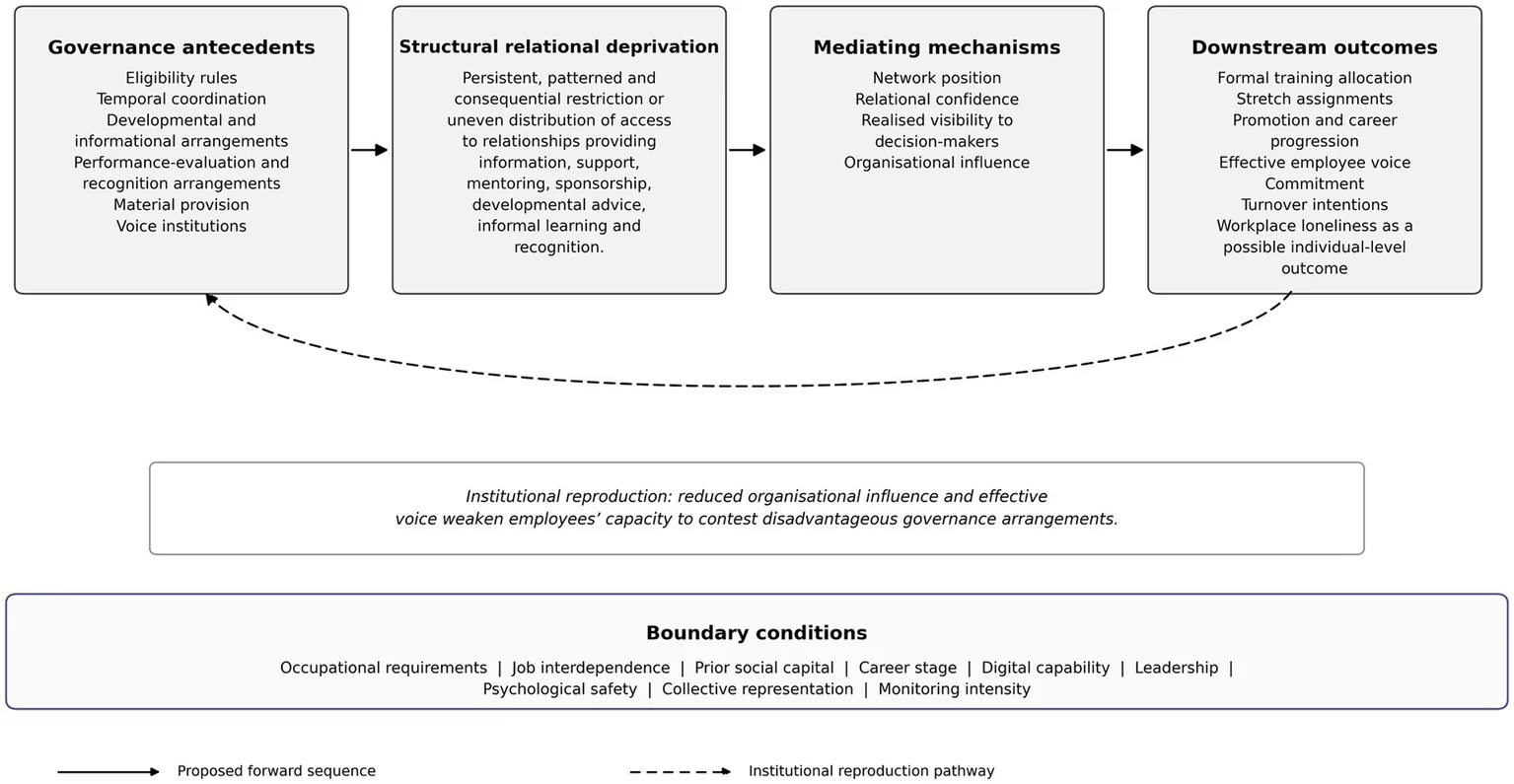
Process model of relational opportunity governance and structural relational deprivation. The model depicts the proposed sequence from governance antecedents through structural relational deprivation and mediating mechanisms to downstream outcomes, together with the institutional reproduction feedback pathway and contextual boundary conditions. Source: Author’s own.

Four features of the definition require emphasis, the first three drawn directly from the sociological literature just discussed. First, following Acker (2006), deprivation refers to a recurring and patterned shortfall produced through routine organisational practice rather than an isolated communication failure or a deliberate act of exclusion: an employee’s inability to reach a colleague on one occasion would not constitute structural deprivation, whereas a persistent arrangement in which remote-dominant employees have limited access to mentoring or career visibility may do so where the pattern can be traced to the organisation of work. Second, following relational inequality theory’s account of social closure, the construct concerns relationships that carry employment value rather than social interaction in general; employees may communicate frequently while remaining excluded from sponsorship or influential decision networks, a distinction supported by evidence that informal communication requires more than technological availability (Begemann et al., 2024) and that successful knowledge-seeking depends on the usability and perceived closeness of a relationship (Keppler and Leonardi, 2023). Third, the construct is inherently distributive, since claims-making processes advantage some groups over others rather than operating uniformly: organisation-wide averages may conceal substantial differences between groups, since the career consequences of home working vary with team norms (Kasperska et al., 2024) and technology perceptions can affect the survival of advice relationships unevenly (Wu et al., 2023). Fourth, the construct is not limited to hybrid work; co-located organisations may also restrict relational opportunity through rigid hierarchy, though hybrid work is theoretically important because it makes relational access more dependent on temporal coordination, digital systems and managerial discretion.

To prevent the construct from describing any degree of ordinary unevenness in workplace relationships, a condition qualifies as structural relational deprivation only where the restriction is, jointly: persistent or recurrent rather than a single episode; patterned across an identifiable group, role configuration or arrangement rather than idiosyncratic to one individual; consequential for role performance, development or employment opportunity rather than merely uncomfortable; and traceable to an identifiable governance condition, such as an eligibility rule, a scheduling practice or a performance process, rather than to unrelated personal circumstance. All four conditions must hold; a shortfall that is patterned but inconsequential, or persistent but untraceable to any governance condition, would not meet the threshold.

Structural relational deprivation is defined independently of workplace loneliness, which is a subjective experience arising when employees perceive their workplace relationships as inadequate relative to their needs (Peplau and Perlman, 1982; Wright et al., 2006). Structural relational deprivation instead concerns the availability and distribution of relational opportunity within the work system: an employee may experience loneliness despite extensive relational access, while another may be excluded from mentoring without reporting loneliness. Loneliness may arise as one consequence of deprivation, but deprivation may also affect learning, development and career progression without producing loneliness at all.

#### 2.3.1 Distinction from adjacent constructs

Table 1 clarifies the boundaries between structural relational deprivation and the closest adjacent constructs, including two, social capital and network disadvantage, that are discussed extensively in the surrounding text but require explicit inclusion here to avoid an inconsistency between the discursive argument and the construct map.

**Table 1:** Differentiation of structural relational deprivation from adjacent constructs.

Construct	Core meaning	Primary level	Nature	Distinctive referent
Structural relational deprivation	Systematic restriction or uneven distribution of access to employment-relevant relationships	Work system, organisation, team or role configuration	Structural	Governance and distribution of relational opportunity
Social capital	Resources embedded in relationships and networks that can be mobilised for advantage	Individual or organisation	Relational and resource-based	The resources themselves, not access to them
Network disadvantage	An employee’s realised, peripheral position within a social or informational network	Individual or network	Primarily objective and positional	Realised network position
Relational coordination	Coordination among interdependent role-holders through shared goals, shared knowledge, mutual respect and timely, accurate and problem-solving communication	Dyad, team or interdependent work process	Relational and structural	Quality of coordination among interdependent roles
Workplace loneliness	Perceived inadequacy of workplace relationships relative to needs	Individual	Subjective	Experienced relational deficiency
Professional isolation	Limited professional contact, interaction or network embeddedness	Individual or network	Primarily objective	Realised contact and professional embeddedness
Workplace exclusion or ostracism	Being ignored, omitted or rejected by others	Individual, dyad or group	Behavioural and perceptual	Interpersonal treatment
Low organisational belonging	A weak sense of acceptance, membership and fit	Individual, with possible shared patterns	Subjective	Organisational membership and identification
Relative deprivation	Perceived disadvantage from an unfavourable comparison	Individual or group	Subjective and comparative	Comparative entitlement and outcomes

Social capital refers to the resources embedded in networks, whereas structural relational deprivation concerns the governed organisational arrangements that shape who can access and develop those networks in the first place; an organisation may possess substantial aggregate social capital while systematically restricting particular groups’ access to it, which is precisely the distributive concern the present construct is designed to capture. Network disadvantage describes an employee’s already-realised position within a network, whereas structural relational deprivation directs attention upstream, to the policies, schedules and managerial practices that produced that position; the two are related as outcome to cause rather than as synonyms. Relational coordination concerns the quality of communication and relationships through which interdependent role-holders coordinate their work, particularly through shared goals, shared knowledge, mutual respect and timely, accurate and problem-solving communication (Gittell, 2002). It shares structural relational deprivation’s concern with organisationally consequential relationships but differs in its principal referent. Relational coordination focuses primarily on the quality of coordination among roles that depend on one another for task accomplishment, whereas structural relational deprivation concerns the governance and distribution of access to a broader range of employment-relevant relationships, including mentoring, sponsorship, informal learning and recognition relationships that may exist beyond immediate task interdependence. Professional isolation concerns actual contact or embeddedness, whereas structural relational deprivation concerns the opportunity structure from which such contact can be developed; employees with established status or strong personal initiative may partly compensate for a depriving arrangement, but their ability to do so does not establish that the underlying work system is equitable. Workplace exclusion and ostracism generally involve acts of interpersonal omission, whereas structural relational deprivation does not require deliberate exclusion and may arise from apparently neutral arrangements, such as leaving hybrid-work decisions largely to managerial discretion, consistent with Acker’s (2006) emphasis on inequality reproduced through routine rather than deliberately discriminatory practice. Relative deprivation rests on a perceived comparison between what an employee receives and believes they are entitled to Crosby (1976), whereas structural relational deprivation may exist before employees recognise the disparity and must therefore be assessed through the organisation of work rather than through dissatisfaction alone.

#### 2.3.2 Illustrative operationalisation

Structural relational deprivation should be operationalised separately from its governance antecedents, mediating mechanisms and downstream consequences. As a system-level condition, it cannot be established adequately through individual perceptions alone. Its empirical assessment should instead examine whether employees or identifiable groups experience persistent and patterned differences in usable access to employment-relevant relationships providing information, practical support, mentoring, sponsorship, developmental advice, informal learning and recognition. Potential indicators therefore include disparities in access to advice and knowledge ties, mentoring and sponsorship relationships, informal learning opportunities, support relationships and recognition-relevant relationships. Such disparities would constitute evidence of structural relational deprivation only where they are persistent or recurrent, patterned across an identifiable group or work arrangement, consequential for employment opportunity or role functioning, and traceable to organisational governance conditions.

The six dimensions identified in Section 2.2 should be measured separately as potential governance antecedents. These include eligibility rules, temporal coordination, developmental and informational arrangements, performance-evaluation and recognition arrangements, material provision and voice institutions. Network position, relational confidence, realised visibility to decision-makers and organisational influence are likewise analytically distinct and should be examined as potential mediators through which structural relational deprivation affects subsequent employment consequences. Formal training allocation, stretch assignments, promotion and career progression, effective employee voice, commitment, turnover intentions and workplace loneliness represent potential downstream outcomes rather than indicators of the construct. Contextual conditions at individual, occupational, organisational and country levels may strengthen, weaken or otherwise condition these relationships. Future construct validation should therefore employ a multilevel design capable of distinguishing structural relational deprivation from its antecedents, mediators, outcomes and boundary conditions and of testing whether it explains variance beyond established constructs such as social capital, professional isolation, organisational support and workplace loneliness.

#### 2.3.3 Disconfirmation criteria

Structural relational deprivation should be rejected or substantially revised if several empirical conditions are observed. First, the construct would be weakened if established alternative explanations, including leadership, individual preference, job interdependence, prior social capital, professional isolation and perceived organisational support, fully account for differences in relational access once measured appropriately. Second, the claim that structural relational deprivation represents a work-system condition would be challenged if proposed indicators of restricted relational access fail to exhibit persistent and patterned differences across identifiable groups, teams, roles or organisational arrangements, or cannot be traced to governance conditions. Third, the proposed governance explanation would require reconsideration if changes to relevant organisational arrangements, such as temporal coordination, developmental and informational provision or material provision, produce no corresponding change in relational access beyond the effects of individual-level interventions. Fourth, the explanatory framework would be weakened if structural relational deprivation fails to predict the proposed mediating mechanisms of network position, relational confidence, realised visibility to decision-makers and organisational influence, or if these mediators do not account for variation in the specified downstream employment outcomes. These criteria make structural relational deprivation open to empirical falsification and prevent it from becoming an encompassing label for any unfavourable relational experience or employment outcome.

### 2.4 Hybrid work as a contested employment arrangement

Hybrid work is commonly described by the number of days employees work away from the employer’s premises, but this measure does not capture the employment relations content of the arrangement: the meaning of two remote days depends on who controls the schedule, whether it can be altered unilaterally, how performance is assessed, which costs are transferred to employees and whether developmental relationships are preserved. The pluralist employee relations tradition recognises that employers and employees have overlapping but not identical interests, and that managerial authority, while necessary for organising work, remains open to contestation where its exercise affects working conditions and employee voice (Ackers, 2014; Kaufman, 2020), a recognition that complements Acker (2006) sociological account of inequality regimes by specifying the institutional mechanisms, consultation, collective bargaining and statutory dispute resolution, through which routine organisational practice can in principle be contested. Hybrid work is therefore not simply a technical HR intervention; it forms part of the continuing negotiation over where, when and under what conditions labour is performed. Employers may use hybrid work to support retention and cost management, while employees may value autonomy and reduced commuting; Bloom et al. (2024) show that these interests can be reconciled under a well-designed arrangement, though interests may also diverge where employers seek closer monitoring and employees seek greater discretion. The arrangement may also redistribute costs, since employees working from home may bear expenses associated with electricity, connectivity and workspace; whether and to what extent South African law requires reimbursement of such costs depends on the applicable contract, policy and collective agreement, and general statements about statutory obligation should be treated with caution outside a dedicated legal analysis.

Voice is especially important in hybrid-work design because managers may not fully understand how scheduling and monitoring affect employees in different circumstances, and consultation can improve both the legitimacy and practical quality of arrangements. O’Brady et al. (2025) show that collective worker voice shaped remote performance-management design, with stronger union and representative institutions better able to secure arrangements balancing performance with employee autonomy. South African evidence adds that voice requires more than formal opportunity: Mohase et al. (2025), Ngubane and Mbokota (2025) and Magwentshu et al. (2026) together show that employees must believe raising concerns will not expose them to career risk before they will use available channels. The South African statutory framework is relevant background rather than a central analytical strand of this article: section 64(4) of the Labour Relations Act 66 of 1995 (Government Gazette, 1995) provides a mechanism in disputes concerning unilateral changes to terms and conditions, while section 186(2) defines unfair labour practices more narrowly and should not be treated as a general remedy for disagreements about hybrid work. The point for present purposes is not the detail of any specific provision, but that voice, consultation and dispute-resolution arrangements form part of relational opportunity governance because they affect employees’ capacity to influence how relational access is distributed, regardless of which specific legal instrument would apply to a given dispute.

Formal policy does not determine the quality of a hybrid arrangement on its own. Bowen and Ostroff (2004) theory of HR-system strength proposes that practices produce consistent employee responses when they send distinctive, consistent and consensual signals, signals that hybrid arrangements can weaken where expectations concerning attendance differ across managers. Nishii et al. (2008) similarly distinguish intended policy from managerial implementation and actual use, a distinction that matters because a policy may formally promise flexibility while local managers implement it through close surveillance; this gap between intention and practice is one of the principal mechanisms through which unequal relational access arises. South African evidence supports this emphasis: Mabaso and Manuel (2024) found that hybrid performance management required more continuous communication than conventional approaches, and Charles et al. (2025) found organisational support strongly associated with engagement in a South African IT company operating hybrid arrangements. Managerial discretion may influence eligibility and developmental access in either direction: Kasperska et al. (2024) show that managers make different promotion decisions according to where an employee works, while Wiatr (2026) shows that responsive managerial presence can be maintained through relationship-building rather than physical co-location alone.

### 2.5 Material access and the South African employment context

Relational opportunity in hybrid work is shaped by material as well as organisational conditions. Employees require reliable electricity, affordable connectivity and suitable workspace to participate consistently in digitally mediated work; these are not private inconveniences detached from employee relations. Where organisations offer formal access to hybrid work without providing the resources required for effective participation, equal policy may produce unequal opportunity, since employees with unstable connectivity or inadequate workspace may be less able to contribute spontaneously or maintain visibility even where their formal eligibility is identical to that of better-resourced colleagues.

The broader labour-market context intensifies this concern. Statistics South Africa’s most recently available Quarterly Labour Force Survey at the time of writing continues to show substantial occupational, gender and population-group inequalities in employment. The survey does not measure hybrid-work eligibility directly and should not be used as though it does, but it establishes that hybrid work is introduced into an employment system in which access to professional, managerial and digitally enabled work is already uneven. The South African context therefore contributes more than local illustration: it shows why relational opportunity must be understood as materially enabled, occupationally differentiated and embedded within a historically unequal employment system, in which a formally uniform hybrid-work arrangement may interact with existing inequalities in occupation, organisational status and access to influential networks. Whether these labour-market inequalities translate into unequal structural relational deprivation specifically is a distinct empirical question that this contextual evidence cannot itself answer, and the propositions in Section 5.6 are framed accordingly as directions for testing rather than as findings already established.

### 2.6 Institutional reproduction: mediators, outcomes and the four-layer model

Structural relational deprivation is proposed to matter because relational access affects employees’ capacity to influence the arrangements governing that access, the specific mechanism of claims-making that relational inequality theory identifies as sustaining organisational inequality once it has arisen (Tomaskovic-Devey and Avent-Holt, 2019). To state the mechanism without circularity, the framework distinguishes four analytical layers, depicted in Figure 1. Governance antecedents comprise eligibility rules, temporal coordination practices, material provision, performance-evaluation and recognition arrangements, developmental and informational arrangements, and voice institutions. Structural relational deprivation refers to a patterned shortfall in employees’ access to relationships providing information, support, developmental advice, sponsorship, informal learning and recognition. Mediators are the realised intermediate consequences of that shortfall, including network position, relational confidence, realised visibility to decision-makers and organisational influence. Outcomes are subsequent employment consequences, including formal training allocation, stretch assignments, promotion, career progression, effective employee voice, commitment and turnover. The model therefore distinguishes access to developmental relationships, which forms part of the construct, from formal organisational development decisions, which are downstream outcomes. Visibility and influence therefore appear once in the model, as mediators between deprivation and its consequences, and not simultaneously as governance dimensions, construct content and predicted outcomes; Proposition 5 in Section 5.6 is written to respect this separation, predicting outcomes that are not already contained within the definition of the construct itself.

Employees occupying central or bridging network positions are more likely to obtain timely information and access influential contacts (Burt, 2004; Seibert et al., 2001), increasing their capacity to negotiate favourable arrangements. Employees at the organisational periphery face the reverse pattern: Keppler and Leonardi (2023) show that employees may recognise who holds valuable knowledge while lacking the relational confidence to approach that person, and Kasperska et al. (2024) show how reduced visibility enters managerial decisions on promotion and remuneration. This article conceptualises the resulting process as an institutional reproduction cycle, the specific sequence through which social closure (Tomaskovic-Devey and Avent-Holt, 2019) operates once relational access has already been restricted: unequal governance produces structural relational deprivation; deprivation reduces the mediators, network position, relational confidence, visibility and influence, that would otherwise allow disadvantaged employees to be recognised and heard; and diminished mediators weaken employees’ capacity to challenge or reshape the governance arrangement, allowing the initial distribution of relational opportunity to persist through subsequent scheduling and developmental decisions. The cycle does not require deliberate exclusion; it may emerge through routine managerial discretion and apparently neutral procedures, as when differences in employees’ technology perceptions contribute to the dissolution of advice relationships without any conscious intention to exclude (Wu et al., 2023), consistent with Acker (2006) account of inequality sustained through ordinary organisational practice. Strong worker-voice institutions can interrupt the cycle, as O’Brady et al. (2025) show for remote-work governance more broadly.

The proposed process differs from an individual resource-loss spiral. Conservation of Resources theory may explain why employees conserve personal effort after repeated relational disappointment (Hobfoll, 1989; Hobfoll et al., 2018), but it operates at the level of individual psychological response and does not by itself explain why unequal access persists as an organisational-level pattern; the institutional reproduction cycle instead centres the sociological relationship between access, organisational influence and the capacity to contest managerial arrangements. Psychological contract and social exchange theories help explain employees’ subsequent individual-level responses: where employees attribute unequal access to organisational decisions, they may interpret the arrangement as a failure of reciprocal obligation even where the formal contract has not changed (Blau, 1964; Cropanzano and Mitchell, 2005; Rousseau, 1989), producing reduced trust as a response to an underlying governance problem rather than as the defining feature of deprivation itself. The theoretical value of the construct lies in identifying a work-system condition that precedes individual experience, giving employee relations research an organisational object that can be measured, compared and redesigned, and allowing it to ask not only whether employees feel connected, but whether they have equitable access to the relationships through which organisational opportunity and influence are realised.

## 3 Research questions

This article examines hybrid work as an employment arrangement through which access to consequential workplace relationships is governed and distributed. The review and theory development are guided by three research questions. First, how do hybrid-work governance arrangements shape the distribution of employment-relevant relational opportunities (RQ1)? Second, which configurations of eligibility, temporal coordination, developmental and informational arrangements, performance-evaluation and recognition arrangements, material provision and voice institutions give rise to structural relational deprivation? (RQ2)? Third, through which organisational mechanisms does structural relational deprivation influence formal training allocation, stretch assignments, promotion, career progression and effective employee voice, and how might these processes contribute to the persistence of unequal hybrid-work governance (RQ3)?

## 4 Research design

### 4.1 Review approach and evidence base

The article adopts a critical integrative review combined with abductive theory development, an approach suited to a problem spanning different constructs, levels of analysis and methodological traditions where the purpose is theoretical integration rather than estimation of an aggregate effect (Cronin and George, 2023; Kunisch et al., 2023). The review integrated scholarship on hybrid and remote work, workplace networks, employee relations, HR implementation and infrastructural conditions to examine how organisations govern and distribute access to employment-relevant relationships.

The evidence base was assembled in two phases. The initial search covered scholarship published between January 2005 and February 2026, with earlier foundational sources retained selectively, followed by a targeted update in June 2026. Scopus and Web of Science were the primary bibliographic databases. Google Scholar supported backward and forward citation tracing and identification of recent and South African scholarship, supplemented by Sabinet, SA ePublications and relevant journal archives. Search terms combined remote- and hybrid-work terminology with employee voice, managerial discretion, mentoring, sponsorship, social capital, relational inequality, HR-system strength, digital inequality, professional isolation, performance evaluation and recognition. The breadth of the search reflected the absence of a common vocabulary for unequal relational access across these literatures (Hiebl, 2023).

The search and screening process was documented through a source inventory, screening log and concept matrix. Titles and abstracts were screened first, followed by full-text assessment where relevant. Following application of the inclusion and exclusion criteria, 43 substantive sources were retained for synthesis. Foundational, methodological, statutory and standards-based sources used to establish the review design, theoretical framing and policy context were recorded separately. Because the study was conducted as a critical integrative review rather than a prospectively registered systematic review, database-level retrieval, deduplication, stage-specific exclusion counts and exact search-execution dates were not retained in a form permitting reliable retrospective reconstruction and are therefore not retrospectively generated. The supplementary review audit reports the available search documentation, source-selection procedure, inclusion and exclusion criteria, recorded exclusion reasons, methodological-appraisal approach and source-selection flow.

Sources were retained where they informed relational access in hybrid work; employee voice, managerial discretion or distributive fairness; workplace networks or relational inequality; differentiation of structural relational deprivation from adjacent constructs; or relevant South African employment-relations and infrastructural conditions. Sources were excluded where hybrid or remote work was incidental, relational or governance processes were peripheral, the level of analysis could not inform the framework, or the source lacked sufficient empirical or conceptual relevance. Research on loneliness outside the employment context was included only where it assisted construct differentiation, and practitioner reports were not used as the principal evidential basis for theoretical claims.

Each substantive source was coded in the concept matrix according to context, design, level of analysis, governance condition, proposed mechanism, outcome and theoretical contribution. No numerical quality score was applied because the evidence base included heterogeneous experimental, longitudinal, network, qualitative and conceptual research. Instead, evidential weight reflected methodological transparency, appropriateness of design and analysis, strength of temporal or causal inference where relevant, and theoretical relevance. Qualitative evidence was used principally to clarify processes and implementation, while conceptual sources were assessed for conceptual clarity and contribution to theory development.

Theory development proceeded abductively through repeated movement between the evidence, established theory and the emerging framework (Cronin and George, 2023). Initial attention to work location, communication and workplace loneliness proved insufficient to explain why employees operating under similar hybrid arrangements could experience different levels of relational access. The analysis therefore shifted towards how relational opportunities are governed and distributed, informing the six governance dimensions, the conceptualisation of structural relational deprivation and the institutional reproduction mechanism. The author’s earlier work on organisational loneliness (Mdhluli, 2026) was treated as an adjacent individual-level conceptual source rather than the foundation of the present system-level framework.

### 4.2 Analytical procedure and rigour

The synthesis proceeded through construct-boundary analysis, comparing structural relational deprivation with social capital, professional isolation, workplace loneliness, exclusion, belonging and relative deprivation and retaining it only where it identified a system-level condition not adequately captured by these established concepts; governance-configuration analysis, treating eligibility, coordination, developmental and informational arrangements, performance-evaluation and recognition arrangements, material provision and voice institutions as an interdependent configuration rather than independent predictors; mechanism mapping, examining how governance arrangements might produce unequal access and developing the institutional reproduction sequence; and adversarial explanation, considering whether leadership, job interdependence, prior social capital, individual preference or digital competence offered sufficient alternative explanations. Evidence that hybrid work can improve autonomy, inclusion or retention was retained as central to the synthesis rather than treated as an exception, so that the framework was required to accommodate beneficial, adverse and mixed outcomes. Findings directly reported in empirical studies were treated as evidence-based patterns, explanations developed by linking separate bodies of scholarship were identified as theoretical inferences, and relationships involving structural relational deprivation and institutional reproduction were presented as propositions requiring future testing, a distinction maintained to avoid presenting conceptual plausibility as established causal evidence.

Because the review was conducted by a single author, it did not permit independent coding or inter-rater agreement; this limitation was addressed, though not eliminated, through a two-pass audit in which the source inventory, inclusion decisions and coding categories were re-audited after an interval of at least 2 weeks, with each source reconsidered against inclusion criteria and evidence challenging the framework actively sought. A final citation audit checked that substantive statements were supported by relevant sources and that sources were not grouped together merely because they addressed a broadly similar topic. The source inventory, screening log and concept matrix are retained by the author and may be supplied to the editor or reviewers on reasonable request.

### 4.3 Ethical and research integrity considerations

The study relied on published research, legislation and publicly available institutional material. No human participants, animals or identifiable personal data were involved; formal ethical approval was therefore not required under the applicable institutional policy. The principal ethical responsibilities concerned the selection and representation of evidence and the potential organisational use of the proposed framework. To address the risk of confirmation bias, the review retained studies reporting positive, negative and mixed outcomes of hybrid work and specified conditions under which the construct should be rejected or revised (Section 2.3.3). The evidence base is weighted towards professional and knowledge-intensive occupations, where hybrid work has been studied most extensively, and the manuscript therefore treats occupational eligibility as an unresolved distributive question rather than assuming the framework applies uniformly across the labour market. Finally, an argument that hybrid work can restrict relational opportunity could be misused to justify compulsory office attendance or intrusive monitoring; the framework does not equate physical presence with meaningful relational access, and its practical implications emphasise diagnosis, coordinated work design, material provision, employee voice and fair access to developmental relationships rather than mandated co-presence.

## 5 Findings and theoretical development

The critical integrative review produced three evidence-based findings and three theoretical outputs. Sections 5.1–5.3 report patterns supported by the reviewed literature: that relational opportunity is shaped by configurations of hybrid-work governance, that structural relational deprivation concerns unequal access to employment-relevant relationships rather than low communication alone, and that this access is distributed unevenly across employees and occupational groups. Sections 5.4–5.6 build on these findings to develop the institutional reproduction mechanism, a typology of relational opportunity governance and testable propositions. Section 5.7 considers rival explanations. This distinction matters because structural relational deprivation has not yet been validated empirically as a separate construct: the review supports the organisational processes from which it is derived, but the full framework remains a theoretical explanation requiring multilevel and longitudinal testing.

Table 2 summarises the principal evidence domains included in the review, the questions addressed by each domain and their role in developing the proposed framework.

**Table 2:** Evidence domains and their role in theory development.

Evidence domain	Question addressed	Evidence retained	Role in theory development
Relational opportunity and workplace networks	Through which relationships do employees obtain information, support, development, recognition and influence?	Social-capital theory, advice and knowledge networks, mentoring, sponsorship, relational confidence and network-position research	Defines employment-relevant relational opportunity and distinguishes access conditions from realised network resources
Hybrid-work design and governance	Which arrangements shape employees’ access to workplace relationships?	Hybrid-work trials, schedule and coordination studies, work-mode configurations and implementation research	Identifies eligibility, temporal coordination and organisational support as governance dimensions
Employee relations, power and voice	Who determines hybrid arrangements, whose interests are represented and how can decisions be contested?	Employment relations pluralism, managerial prerogative, employee voice, consultation and collective bargaining	Positions hybrid work as a contested employment arrangement and explains power, legitimacy and contestation
HR implementation, visibility and development	How do formal policies become uneven practices and employment outcomes?	HR-system strength, HR-process research, managerial discretion, performance-evaluation and proximity-bias studies	Explains how formally neutral arrangements may generate unequal developmental and career access
Digital communication and material participation	How do technologies, norms and infrastructure condition relational access?	Communication affordances, sociomateriality, technology-use differences, digital inequality and South African infrastructure research	Establishes the sociotechnical and material conditions required for substantive participation
Adjacent constructs and outcomes	How does structural relational deprivation differ from existing relational experiences and positions?	Social capital, network disadvantage, professional isolation, workplace loneliness, belonging, exclusion and relative deprivation	Establishes construct boundaries and positions loneliness as one possible outcome rather than the focal phenomenon

### 5.1 Governance configurations shape relational opportunity

The consequences of hybrid work cannot be explained by the number of days employees work away from an organisational workplace. Lauring and Jonasson (2025) show that hybrid work varies across spatial location, communication modality and time, while Handke et al. (2024) emphasise that hybrid teams operate through shifting geographical configurations that create distinctive coordination demands; hybrid-work intensity is therefore a limited indicator unless considered alongside the rules and resources through which the arrangement is implemented. Bloom et al. (2024) randomised trial of 1,612 employees demonstrates that positive outcomes are possible where a coordinated arrangement is predictable and institutionally supported, without establishing that hybrid work is uniformly beneficial. South African evidence points the same way: Charles et al. (2025) found organisational support strongly associated with engagement in a Western Cape IT company operating hybrid arrangements, and Mabaso and Manuel (2024) found that clear performance expectations, continuous communication, trust and consistent standards across locations were central to employees’ and managers’ experience of remote and hybrid performance management. Begemann et al. (2024) further demonstrate that informal communication depends on interacting technological, social and individual conditions rather than technology or location alone, illustrating that the governance dimensions identified in Section 2.2 operate as an interdependent configuration: employee discretion over location may preserve autonomy while producing limited relational benefit if team schedules are poorly coordinated, while coordinated attendance may increase access to colleagues but remain inequitable if employees have little influence over its terms. This first finding answers RQ1: hybrid-work governance shapes relational opportunity through the combined effects of eligibility, coordination, developmental and informational arrangements, performance-evaluation and recognition arrangements, material provision and voice institutions with work location establishing the context in which these arrangements operate rather than determining their relational consequences independently.

### 5.2 Structural relational deprivation arises from unequal access

The synthesis indicates that the relational consequences of hybrid work are better understood as differences in the distribution of relational opportunity than as a uniform consequence of working away from a central workplace. Employment-relevant relational opportunity cannot be inferred from communication frequency. Yang et al. (2022) provide large-scale evidence for this distinction: following the move to firm-wide remote work at Microsoft, employees’ collaboration networks became more static and siloed, with fewer connections across organisational boundaries, even as digital activity remained extensive. Keppler and Leonardi (2023) explain why formal access to expertise may not become usable relational access, showing that remote and hybrid employees may know where relevant knowledge resides while still lacking sufficient relational confidence to approach the person who holds it, and Wu et al. (2023) similarly found that advice-seeking relationships were less likely to be maintained where advice seekers and providers differed in their perceptions of communication-technology usability, an effect stronger for those who worked remotely more often. Begemann et al. (2024) add that organisations’ formal initiatives to stimulate informal interaction did not automatically restore spontaneous or useful relational exchange. Taken together, these studies indicate that a substantive relational opportunity requires an identifiable and reasonably accessible relationship, an interaction that permits access to the resource required, and an effort threshold that does not require substantially more from one group of employees than from others who obtain the same resource routinely. Structural relational deprivation arises where these conditions are restricted or unevenly distributed: an employee may communicate frequently while lacking access to sponsors or decision-makers, or may experience little social interaction while retaining reliable access to the relationships needed to perform and progress. Employees may compensate for a restrictive opportunity structure through prior relationships or exceptional relational skill, but such compensation does not establish that the arrangement is equitable; where some employees must invest substantially more effort to obtain what others receive routinely, the organisation has effectively transferred responsibility for a structural shortfall to the individual. This second finding answers the first part of RQ2: structural relational deprivation is produced by the systematic restriction or unequal distribution of usable access to employment-relevant relationships, not by low communication in itself.

### 5.3 Structural relational deprivation is patterned rather than uniform

Employees enter hybrid arrangements with different levels of occupational status, prior relational capital, schedule control and influence over organisational decisions, so that formally identical policies can produce materially different opportunities.

*Eligibility and occupational position.* Eligibility for hybrid work is shaped substantially by occupational requirements: professional and knowledge-intensive roles are generally more amenable to remote performance than roles requiring physical service delivery, making hybrid-work eligibility itself a distributive feature of the employment system, even though most empirical research concentrates on employees who already qualify. Statistics South Africa’s Quarterly Labour Force Survey (Statistics South Africa, 2026) does not measure hybrid-work eligibility, but it documents the occupational, gender and population-group differentiation of the South African labour market within which access to professional work is allocated; any claim that hybrid work reproduces racial or gender inequality must be tested directly rather than inferred from labour-market statistics alone, but existing inequalities make distribution a necessary empirical question. The eligibility divide also extends to on-site employees who sustain the functions on which hybrid arrangements depend; the possibility that these employees carry additional coordination demands is theoretically relevant but not yet established by the reviewed evidence and should be investigated as a future research question.

*Visibility and formal development allocation.* Kasperska et al. (2024) found experimentally that employees described as working from home were less likely than comparable office-based employees to be selected for promotion, salary increases and formal training. These disadvantages were smaller where home working was common within the team, indicating that location-related career judgements depend on managerial and team norms rather than arising inevitably from remote work. South African evidence points in the same direction without establishing a location penalty directly: Mabaso and Manuel (2024) found that transparent performance systems can reduce reliance on physical observation, although their study does not determine whether such practices eliminate unequal visibility or formal development allocation.

*Material participation.* Formal eligibility for hybrid work does not guarantee the material capacity to participate effectively. Ramantswana (2025) study of South African teleworkers identified flexibility and autonomy alongside difficulties associated with unreliable electricity and inconsistent organisational support in a resource-constrained environment that should not be generalised to every South African workplace, but which demonstrates that two employees governed by the same formal policy may have different access to meetings and development depending on their infrastructure.

*Employee voice.* O’Brady et al. (2025) show that collective worker voice shaped remote performance-management systems independently of technology, while Mohase et al. (2025) found that psychological safety mediated the relationship between inclusive leadership and employee voice among South African remote and hybrid employees. Voice occupies a dual position in the wider framework. Voice institutions constitute a governance antecedent, whereas effective employee voice is treated as a downstream capacity through which employees may contest the arrangements shaping relational access. Access to managers and representative channels may facilitate this process, but voice is not included in the content of structural relational deprivation. This third finding answers the remainder of RQ2: structural relational deprivation is more likely where eligibility, temporal coordination, developmental and informational arrangements, performance-evaluation and recognition arrangements, material provision and voice institutions are governed unevenly. To clarify the analytical boundaries of structural relational deprivation and distinguish the construct from the mechanisms and consequences surrounding it, Table 3 specifies its proposed indicators, mediating mechanisms, potential downstream outcomes and relevant contextual and boundary conditions.

**Table 3:** Analytical specification of structural relational deprivation.

Analytical component	Definition/Function	Proposed indicators or elements	Analytical level
Structural relational deprivation	Focal structural condition comprising persistent, patterned and consequential restriction or uneven distribution of usable access to employment-relevant relationships	Unequal or restricted access to relationships providing information, practical support, mentoring, sponsorship, developmental advice, informal learning and recognition	Work system, organisation, team or role configuration
Mediating mechanisms	Relational positions and capacities through which structural relational deprivation is proposed to affect subsequent consequences	Network position; relational confidence; realised visibility to decision-makers; organisational influence	Individual and network
Potential downstream outcomes	Employment and individual consequences proposed to arise through the mediating mechanisms	Formal training allocation; stretch assignments; promotion and career progression; effective employee voice; commitment; turnover intentions; workplace loneliness	Primarily individual and employment-decision level
Contextual and boundary conditions	Conditions that may strengthen, weaken or alter the proposed relationships	Occupational requirements; job interdependence; prior social capital; career stage; digital capability; leadership; psychological safety; collective representation; monitoring intensity; material and infrastructural conditions; broader organisational and country context	Individual, occupational, team, organisational and country

The distinction is important because structural relational deprivation is not defined by its consequences. Restricted or uneven relational access constitutes the focal structural condition. Network position, relational confidence, realised visibility and organisational influence represent mechanisms through which that condition may become consequential. Employment and individual consequences occur further downstream. Contextual and boundary conditions may alter the strength or form of these relationships without constituting structural relational deprivation themselves.

### 5.4 Theoretical development: institutional reproduction of unequal relational access

The reviewed studies show that governance configurations distribute relational opportunity unevenly, but they do not provide direct longitudinal evidence that this inequality reproduces itself over time; the institutional reproduction mechanism developed here is therefore a theoretical inference connecting evidence on workplace networks, visibility, voice and managerial implementation. Employees with established relationships and central network positions are generally better placed to obtain timely information and access influential contacts (Burt, 2004; Seibert et al., 2001). In hybrid settings, Keppler and Leonardi (2023) show that relational confidence affects whether employees approach potential knowledge providers, Kasperska et al. (2024) show that work location can influence managerial decisions on promotion and remuneration, and O’Brady et al. (2025) show that employees’ collective capacity to shape remote-management systems depends on the strength of worker voice. Considered together, these studies support a plausible sequence: unequal governance restricts access to information and recognition; restricted access reduces employees’ visibility and organisational influence; and employees with less influence have weaker capacity to contest the eligibility rules, schedules or managerial practices that produced the disadvantage, so that the original arrangement may persist through subsequent decisions even where exclusion was not deliberate. This process can be summarised as:

*Unequal relational opportunity governance → structural relational deprivation → reduced mediators (network position, relational confidence, realised visibility to decision-makers and organisational influence) → weaker capacity to exercise effective voice → persistence of the governance arrangement.*

The mechanism does not assume that employees are passive; they may seek mentors, build digital networks or use formal voice channels, but such action requires time, relational confidence and organisational responsiveness, and may be easier for employees who already possess status or network resources, so that individual agency can mitigate disadvantage without correcting the work-system conditions that produced it. Nor is the cycle inevitable: transparent allocation criteria, accessible mentoring, psychological safety and effective representative voice may interrupt it, as O’Brady et al. (2025) and Mohase et al. (2025) demonstrate for worker representation and inclusive leadership respectively, although the full reproduction mechanism remains to be tested longitudinally.

Figure 1 illustrates the proposed four-layer model and institutional reproduction mechanism.

### 5.5 A typology of relational opportunity governance

The six governance dimensions and the two typology axes operate at different analytical levels. The six dimensions identify the substantive domains through which relational opportunity is governed: eligibility, temporal coordination, developmental and informational arrangements, performance-evaluation and recognition arrangements, material provision and voice. The two axes do not replace or collapse these dimensions. Rather, they characterise the broader configuration within which the six dimensions operate. Governance participation captures the extent to which employees participate in decisions across these domains, whereas institutional relational provision captures the extent to which the organisation provides structures and resources that enable substantive relational access. The resulting typology therefore offers a parsimonious means of comparing governance configurations while retaining the six dimensions as distinct domains of relational opportunity governance.

The synthesis supports a provisional typology organised around these two axes. Governance participation concerns whether the rules governing hybrid work are determined primarily by management or shaped through meaningful direct and representative employee voice. Institutional relational provision concerns the extent to which the organisation provides the coordination, developmental relationships, material resources and recognition arrangements required to sustain substantive relational opportunity.

Governance participation ranges from management-directed arrangements, in which employees have limited influence over the design and implementation of hybrid work, to co-governed arrangements, in which employees and their representatives participate meaningfully in shaping the governing rules. Institutional relational provision ranges from low provision, where employees remain largely responsible for establishing and maintaining their own relational access, to high provision, where the organisation provides coordinated schedules, developmental infrastructure, material provision and transparent recognition processes.

The intersection of these dimensions produces four ideal-type configurations: management-directed, low institutional relational provision; co-governed, low institutional relational provision; management-directed, high institutional relational provision; and co-governed, high institutional relational provision. These configurations are analytical rather than mutually exclusive descriptions of whole organisations. Different teams may occupy different configurations, and an arrangement may move between configurations as governance practices, resources and employee representation change.

Operational autonomy and monitoring intensity are treated as cross-cutting modifiers rather than as defining dimensions of the typology. Operational autonomy may be high or low within either management-directed or co-governed arrangements because employees may possess considerable day-to-day discretion without having meaningful influence over the rules governing that discretion. Monitoring may support coordination and accountability, but extensive or opaque surveillance may weaken trust and employee voice regardless of the level of institutional relational provision. Future research should therefore test whether these modifiers strengthen or weaken the relationships predicted for each configuration (Table 4).

**Table 4:** Types of relational opportunity governance in hybrid work.

Configuration	Governance conditions	Defining characteristics	Predicted relational pattern	Principal employee-relations risk
Management-directed, low institutional relational provision	Low governance participation × low institutional relational provision	Management determines eligibility, schedules and performance arrangements with limited employee influence. Coordination, mentoring, material provision and relational maintenance are left largely to employees.	High likelihood of uneven access to information, mentoring, sponsorship, recognition and influential networks, particularly among newcomers and organisationally peripheral employees.	Managerial authority is exercised without adequate employee voice, while responsibility for maintaining relational access is individualised.
Co-governed, low institutional relational provision	High governance participation × low institutional relational provision	Employees and their representatives participate in shaping hybrid-work rules, but organisational investment in coordination, mentoring, infrastructure and relational support remains limited.	Employees may identify and contest relational inequalities, but participation alone may not overcome weak material and developmental provision.	Employee voice may become symbolic where consultation is not accompanied by resources, implementation capacity or institutional support.
Management-directed, high institutional relational provision	Low governance participation × high institutional relational provision	The organisation provides coordinated schedules, mentoring, structured interaction, material resources and transparent performance processes, but employees have limited influence over their design.	Relational access may improve, although arrangements may be rigid, compulsory or insufficiently responsive to employees’ circumstances.	Organisational connection is prioritised without adequate consent, participation or contestability.
Co-governed, high institutional relational provision	High governance participation × high institutional relational provision	Direct and representative employee voice shape hybrid-work governance, supported by coordinated schedules, developmental infrastructure, material provision and transparent performance-evaluation and recognition arrangements.	Relational opportunity is most likely to be accessible, usable and equitably distributed across groups and work locations.	Consultation may become procedural or administratively burdensome unless decision rights, accountability and implementation responsibilities are clear.

### 5.6 Propositions

The following propositions translate the framework into testable claims. Consistent with the four-layer model in Section 2.6, Propositions 1 to 4 concern the relationship between governance antecedents and structural relational deprivation, Proposition 5 concerns the relationship between deprivation and its mediators, and Proposition 6 concerns the relationship between those mediators and downstream outcomes, so that no proposition predicts a variable already contained within the definition of the predictor.

**Proposition 1** Hybrid-work intensity will be more strongly associated with structural relational deprivation where temporal coordination is weak and developmental and informational arrangements are limited than where these governance conditions are well managed, holding eligibility, performance-evaluation and recognition arrangements, material provision and voice institutions constant.

**Proposition 2.** Holding governance participation constant, configurations characterised by high institutional relational provision will be associated with lower structural relational deprivation than configurations characterised by low institutional relational provision. Holding institutional relational provision constant, co-governed configurations will be associated with lower structural relational deprivation than management-directed configurations. Structural relational deprivation is therefore expected to be lowest under co-governed, high institutional relational provision arrangements and highest under management-directed, low institutional relational provision arrangements.

**Proposition 3.** Managerial discretion will be more strongly associated with structural relational deprivation where hybrid-work policies are implemented inconsistently and where performance, recognition and career decisions depend substantially on informal managerial familiarity rather than transparent and location-neutral evaluation criteria.

**Proposition 4.** The gap between formal eligibility for hybrid work and substantive relational opportunity will be greater where employees lack adequate material resources or coordinated access to relevant colleagues, and this gap will be more pronounced in resource-constrained employment settings than in well-resourced ones.

**Proposition 5.** Structural relational deprivation will be associated with lower network centrality, lower relational confidence, reduced realised visibility to decision-makers and lower organisational influence, after accounting for prior social capital, professional isolation and perceived organisational support.

**Proposition 6a.** Reduced network centrality, relational confidence, realised visibility to decision-makers and organisational influence will predict less favourable formal development and employment decisions, including lower allocation to training and stretch assignments and weaker prospects for promotion and career progression.

**Proposition 6b.** Reduced organisational influence will also predict weaker effective employee voice concerning hybrid-work governance, which will increase the likelihood that governance arrangements associated with structural relational deprivation persist.

**Proposition 6c.** The indirect relationship between structural relational deprivation and the persistence of disadvantageous governance arrangements through reduced relational mediators and effective employee voice will be weaker where allocation criteria are transparent, psychological safety is high and collective representation is effective.

### 5.7 Rival explanations and boundary conditions

*Leadership* may account for substantial variation in employees’ relational access, since managers influence availability, mentoring and psychological safety; Mohase et al. (2025) and Ngubane and Mbokota (2025) show inclusive leadership and relational competencies supporting employee voice through psychological safety. Leadership is an important governance condition and potential moderator, but it does not replace the structural explanation, since managers exercise discretion within policies, resource constraints and voice institutions that may either support or limit equitable practice.

*Job interdependence and occupational requirements* shape whether hybrid work is feasible and how strongly fragmented schedules affect relational access; Handke et al. (2024) caution that hybrid teamwork must be analysed in relation to team configuration and task demands rather than location alone. These factors are important boundary conditions, but they do not explain whether employees in comparable roles receive equitable access to developmental relationships and voice.

*Prior social capital and individual agency* explain why employees with established networks or stronger relational confidence may convert similar formal opportunities into different outcomes (Keppler and Leonardi, 2023; Wu et al., 2023), without establishing that the underlying opportunity structure is equitable, particularly where overcoming restricted access requires disproportionate personal effort.

*Technology quality and digital capability* can widen or weaken access to information and colleagues (Begemann et al., 2024; Wu et al., 2023), but these effects are embedded in relational norms and organisational practice and are therefore incorporated within material and developmental governance rather than treated as a complete alternative explanation.

*Individual preference* for more or less workplace interaction may shape how employees experience a given governance configuration, but preference does not account for whether the configuration itself distributes relational opportunity fairly across employees who differ in status, resources and influence.

## 6 Discussion

### 6.1 From work location to relational opportunity governance

The review resolves the apparently contradictory evidence on hybrid work by showing that work location alone is not an adequate explanation of relational or employment outcomes: hybrid work can improve satisfaction and retention without impairing performance when coordinated and institutionally supported (Bloom et al., 2024), while employees who work remotely with similar frequency may nevertheless experience substantially different conditions depending on spatial, modal and temporal configuration (Handke et al., 2024; Lauring and Jonasson, 2025). The relevant theoretical question is therefore not whether hybrid work is beneficial or harmful, but how the arrangement governs employees’ access to relationships through which information, support, development, recognition, influence and voice are obtained. This reframing advances employee relations scholarship by treating hybrid work as a substantive feature of the employment relationship: decisions on location, monitoring and performance-evaluation and recognition arrangements allocate organisational benefits, obligations and risks, and O’Brady et al.’s (2025) evidence that collective voice shapes remote performance-management systems shows these arrangements to be outcomes of institutional power and contestation, not merely technical design. Employee voice is therefore not an external moderator added after the hybrid system has been designed; it is part of the governance arrangement through which relational opportunity is allocated.

### 6.2 Structural relational deprivation as an organisational inequality process

Structural relational deprivation extends the analysis of workplace inequality by locating unequal relational access within the organisation of work. This is consistent with Acker (2006) conception of organisational inequality regimes as interrelated practices through which inequalities are maintained, including practices that appear routine or formally neutral, and draws on relational inequality theory’s account of how organisational resources are distributed through processes such as social closure and claims-making (Tomaskovic-Devey and Avent-Holt, 2019). SRD narrows these broader perspectives to a specific organisational resource: access to the relationships through which employees become informed, supported, developed and recognised.

This perspective is important because formally neutral hybrid-work arrangements may generate unequal relational opportunity without explicitly excluding particular employees. Eligibility rules, scheduling practices, developmental arrangements, performance and recognition processes, material provision and voice institutions may shape who can participate in employment-relevant relationships and under what conditions. SRD therefore provides a mechanism for examining how routine organisational arrangements may contribute to the reproduction of workplace inequality even where employees formally possess similar access to hybrid work.

SRD remains conceptually distinct from workplace loneliness. Workplace loneliness concerns the perceived inadequacy of relationships relative to an employee’s relational needs, whereas SRD concerns the structural distribution of access to relationships carrying employment value. Employees may therefore feel socially connected while having limited access to mentoring, sponsorship or recognition-relevant relationships; by contrast, they may experience loneliness despite extensive professional access. This distinction also differentiates the present framework from the author’s earlier work on organisational loneliness (Mdhluli, 2026), which examined how frequent digitally mediated interaction can coexist with an individual experience of insufficient meaningful connection. Workplace loneliness is treated here as one possible downstream response and is neither necessary nor sufficient evidence of SRD.

### 6.3 Institutional reproduction, visibility and employee voice

Acker (2006) inequality-regimes approach and relational inequality theory’s emphasis on social closure and unequal claims-making (Tomaskovic-Devey and Avent-Holt, 2019) provide the theoretical basis for the institutional reproduction mechanism outlined in Section 5.4. Within this process, unequal governance restricts access to information and influential contacts; restricted access reduces visibility and organisational leverage; and employees with less influence are less able to contest the arrangements that produced their disadvantage, so that the original governance arrangement may persist through subsequent decisions even where no actor intended to exclude particular employees. The reviewed studies support individual links in this sequence without yet establishing the complete longitudinal process: Kasperska et al. (2024) demonstrate the visibility-to-career-outcome link, while O’Brady et al. (2025) and Mohase et al. (2025) demonstrate that employees’ ability to influence hybrid-work systems depends on both institutional representation and the relational safety required to speak. The mechanism is conditional rather than inevitable; transparent allocation criteria and effective representative voice may interrupt the cycle, though compensatory action may require greater effort from employees who already possess fewer organisational resources, leaving the underlying arrangement unchanged.

### 6.4 The South African theoretical contribution

The South African context strengthens the framework in three respects. First, it demonstrates that relational opportunity is materially constituted: Ramantswana (2025) findings indicate that South African teleworking is shaped by infrastructural constraints and uneven organisational support, conditions that should be analysed as features of hybrid-work governance rather than private inconveniences. Second, it foregrounds occupational eligibility: Statistics South Africa’s labour-force data document substantial occupational, gender and population-group differentiation which, while not establishing causation, show that hybrid arrangements enter an employment system in which professional status and access to resources are already uneven, so that these patterns should be investigated as possible distributive conditions rather than assumed neutral. Third, it highlights the importance of direct and collective employee voice, since South African evidence shows that inclusive leadership and psychological safety shape employees’ willingness to speak in remote and hybrid settings (Mohase et al., 2025; Ngubane and Mbokota, 2025), reinforcing the argument that formally available flexibility does not produce equitable relational opportunity unless employees can participate safely in its implementation. The framework accordingly does not prescribe compulsory office attendance as a remedy for relational inequality: physical co-presence may increase encounter opportunities, but it does not guarantee access to mentors or effective voice, and attendance without coordination may impose costs without ensuring that relevant relationships are available. The appropriate employee relations response is to diagnose how relational opportunity is distributed and redesign the governance arrangement where patterned restrictions are identified.

## 7 Limitations and strengths

The principal limitation is that this article develops theory rather than testing the proposed construct: structural relational deprivation has not been validated as an empirically distinct work-system condition, and the institutional reproduction pathway has not been demonstrated longitudinally. The review identifies evidence for the governance conditions and intermediate processes from which the framework is developed but cannot establish the prevalence of deprivation or confirm the complete causal sequence. The critical integrative design is concept-driven rather than exhaustive, and the review was conducted by one author without independent coding, a limitation addressed but not eliminated by the two-pass audit described in Section 4.2. Hybrid-work research remains concentrated in professional and knowledge-intensive occupations, so the framework has a stronger evidential foundation for hybrid-eligible employees than for frontline and precarious workers and claims about the burdens carried by hybrid-ineligible employees should remain propositions for future investigation. The South African evidence is similarly concentrated in particular organisations and sectors and should not be generalised across the labour market without further evidence (Mabaso and Manuel, 2024; Mohase et al., 2025; Ngubane and Mbokota, 2025; Ramantswana, 2025). Finally, the framework involves processes at different levels of analysis, since governance resides primarily at organisational level, deprivation may be organisation-wide or concentrated in identifiable groups, and outcomes such as loneliness and turnover occur at individual level, meaning a single-source, cross-sectional survey would not suffice to test these cross-level relationships. The article’s strengths lie in its conceptual discipline: the construct is defined independently of its antecedents and of workplace loneliness, is subject to explicit disconfirmation criteria, and is developed through a documented audit trail that retained disconfirming and mixed evidence rather than evidence supporting the framework alone.

## 8 Recommendations for future research

Future research should establish the empirical distinctiveness and explanatory value of structural relational deprivation. Measurement should capture persistent and patterned disparities in usable access to information, support, mentoring, sponsorship, informal learning and recognition, while assessing governance antecedents and proposed mediators separately. Multilevel designs should determine whether these disparities cluster meaningfully within work arrangements, teams or role configurations, whether governance conditions are sufficiently shared to support aggregation, and whether SRD demonstrates discriminant validity against social capital, professional isolation, organisational support and workplace loneliness (González-Romá and Hernández, 2023). Longitudinal research is needed to test institutional reproduction by establishing whether SRD precedes reductions in network position, relational confidence, realised visibility to decision-makers and organisational influence, and whether weaker effective voice subsequently predicts the persistence of disadvantageous governance arrangements. The proposed typology should be examined through comparative case studies or qualitative comparative analysis to determine whether particular combinations of governance participation and institutional relational provision are associated with lower or higher SRD and whether governance participation and operational autonomy remain empirically distinct. South African research should examine the framework across public administration, higher education, financial services and healthcare, investigating demographic inequalities through organisational processes rather than inferring them from national labour-force patterns. Comparisons between hybrid-eligible and on-site employees within the same organisation could further establish whether differences emerge in workload, coordination demands and voice. Intervention studies should examine whether governance redesign produces stronger relational and employment outcomes than interventions directed primarily at individual employees, including effects on network reach, career progression and workplace loneliness.

## 9 Practical, managerial and policy implications

Organisations should assess the distribution of relational opportunity rather than relying on attendance rates or communication volume. Leaders and HR or People and Culture functions should periodically audit eligibility, temporal coordination, developmental and informational arrangements, performance evaluation and recognition, material provision and voice institutions. Eligibility decisions should use transparent, job-relevant criteria, with managerial discretion documented and reviewable. Temporal coordination should focus on work that genuinely requires shared interaction (Lauring and Jonasson, 2025), while mentoring, sponsorship and developmental access should not depend on incidental proximity. Performance processes should distinguish contribution from physical or digital visibility and recognise relational contributions such as mentoring (Kasperska et al., 2024; Mabaso and Manuel, 2024). Technology and monitoring practices should likewise be assessed for relational consequences, proportionality and fairness, with adequate material provision where remote participation is expected (Begemann et al., 2024; Wu et al., 2023).

Employee Relations specialists, trade unions and employee representatives have a particular role in ensuring that hybrid-work governance incorporates meaningful voice. Consultation should extend beyond work-location preferences to eligibility criteria, scheduling, monitoring, performance processes, developmental access and material provision. Direct employee participation should not substitute for representative voice where established collective structures exist (O’Brady et al., 2025; Mohase et al., 2025). These mechanisms may help identify patterned relational disadvantage before it becomes embedded in routine organisational practice.

At policy level, relational opportunity may also inform psychosocial risk management. ISO 45003 (International Organisation for Standardization, 2021) provides guidance on psychosocial risks associated with work organisation, and relational-opportunity audits could complement existing assessment processes where employee privacy is protected. The framework does not establish a new legal cause of action or general statutory right to hybrid work. In South Africa, changes to hybrid arrangements remain subject to applicable contracts, organisational policies, collective agreements and the existing statutory framework. Compulsory office attendance should therefore not be treated as the default remedy for relational difficulty; the objective is equitable and usable relational opportunity rather than maximised physical presence.

## 10 Contribution and value of the study

The article makes three connected theoretical contributions. The first is relational opportunity governance, explaining how organisations enable, restrict and distribute employees’ access to employment-relevant relationships, moving hybrid-work analysis beyond location and frequency to the employment arrangement through which relational opportunity is produced and contested. The second is structural relational deprivation, a system-level construct that supplies an organisational referent examinable independently of whether employees report loneliness, connecting relational access to broader theories of organisational inequality while retaining a specific domain for empirical validation. The third is the institutional reproduction mechanism, drawing on inequality-regimes and relational-inequality scholarship (Acker, 2006; Tomaskovic-Devey and Avent-Holt, 2019) to explain how restricted access may reduce visibility and thereby allow disadvantageous governance arrangements to persist, transforming the framework from a static account of unequal access into a process explanation of how such inequality becomes embedded in routine organisational decisions. The governance typology and South African analysis elaborate these contributions rather than constituting separate claims of novelty, specifying how employee influence and institutional provision combine in practice and demonstrating why relational opportunity must be understood as materially enabled, occupationally differentiated and institutionally contestable. Together, the three contributions reposition hybrid work as a question of governance, power and distributive fairness: hybrid work does not merely alter where employees perform their duties but can alter who has access to the relationships through which organisational knowledge, development, recognition, influence and voice are realised.

## 11 Conclusion

The relational consequences of hybrid work cannot be inferred from office attendance or remote-work frequency alone; they depend on how organisations govern eligibility, temporal coordination, developmental and informational arrangements, performance evaluation and recognition, material provision and voice. These governance arrangements may generate structural relational deprivation when relational opportunity is distributed unevenly across employees or groups. Its consequences may unfold through reduced network position, relational confidence, realised visibility and organisational influence, with implications for development, career progression, effective voice and other employment outcomes. Unequal relational opportunity may also become self-reproducing when diminished organisational influence and effective voice weaken employees’ capacity to challenge the arrangements that sustain their disadvantage. The central employee-relations task is therefore neither to abandon flexibility nor to romanticise physical co-presence, but to govern relational opportunity deliberately, transparently and fairly. The central question for hybrid-work research is not simply where employees work, but how organisations govern who gains substantive access to the relationships through which work, development, recognition and opportunity are realised.

## Data Availability

The original contributions presented in the study are included in the article/Supplementary material, further inquiries can be directed to the corresponding author.
