# Supplementary material for: Governing relational opportunity in hybrid work: structural relational deprivation and the reproduction of workplace inequality
Source: Front Sociol. 2026 Sep 30;11:1955187. doi: 10.3389/fsoc.2026.1955187 (PMC13661433; doi:10.3389/fsoc.2026.1955187)
Supplement: Supplementary file 1 [file Supplementary_file_1.docx]

**Supplementary Material**

This supplementary audit documents the review procedures that can be verified from the concurrent source inventory, screening log and concept matrix. The study was designed as a critical integrative review combined with abductive theory development rather than as a prospectively registered systematic review. Accordingly, information that was not retained prospectively is not reconstructed retrospectively.

# S1. Search strategy and coverage

The evidence base was assembled in two phases. The initial search covered research articles published between January 2005 and February 2026, while earlier foundational sources were retained selectively. Following refinement of the governance-centred focus, a targeted search update was conducted in June 2026.

Scopus and Web of Science were the primary bibliographic databases. Google Scholar was used for backward and forward citation tracing and to identify recent and South African scholarship. Searches were supplemented through Sabinet, SA ePublications and relevant journal archives. The journal-archive searches included the African Journal of Employee Relations, SA Journal of Human Resource Management and SA Journal of Industrial Psychology.

Search terms combined remote- and hybrid-work terminology with employee voice, managerial discretion, mentoring, sponsorship, social capital, relational inequality, HR-system strength, digital inequality, professional isolation, performance evaluation and recognition. The search was deliberately broad because the literatures relevant to unequal relational access do not use a common vocabulary.

Exact search-execution dates, database-level retrieval counts and deduplication counts were not retained prospectively in a form permitting reliable reconstruction. They are therefore not reported retrospectively.

# S2. Source selection and eligibility

Screening proceeded through assessment of titles and abstracts and, where potentially relevant, full texts. Sources were retained where they informed one or more of the following: relational access in hybrid work; employee voice, managerial discretion or distributive fairness; workplace networks or relational inequality; differentiation of structural relational deprivation from adjacent constructs; or South African employment-relations and infrastructural conditions relevant to the framework.

Sources were excluded where hybrid or remote work was incidental, relational or governance processes were peripheral to the analysis, the level of analysis could not inform the proposed framework, or the source lacked sufficient empirical or conceptual relevance to the theoretical problem. Research on loneliness outside the employment context was included only where it assisted construct differentiation. Practitioner reports were not used as the principal evidential basis for theoretical claims.

Following application of these criteria, 43 substantive sources were retained for the integrative synthesis. Foundational, methodological, statutory and standards-based sources used to establish the review design, theoretical framing and policy context were recorded separately and were not counted among the 43 substantive sources.

# S3. Methodological appraisal and concept matrix

Each retained substantive source was coded in a concept matrix according to context, research design, level of analysis, relevant governance condition, proposed mechanism, outcome and contribution to theory development.

No numerical methodological-quality score was imposed because the evidence base included heterogeneous experimental, longitudinal, network, qualitative and conceptual research that could not appropriately be ranked through a single methodological hierarchy. Evidential weight instead reflected methodological transparency, appropriateness of design and analysis, strength of temporal or causal inference where relevant, and theoretical relevance. Studies capable of stronger temporal or causal inference were given greater weight when directional relationships were evaluated. Qualitative evidence was used principally to clarify relational processes, meaning and implementation, while conceptual sources were assessed for conceptual clarity, theoretical relevance and contribution to construct development.

# S4. Source-selection flow

Because exact stage-specific numerical screening counts were not retained prospectively, the selection process is presented as a non-numerical audit trail rather than as a numerical PRISMA flow diagram.

Identification of potentially relevant sources

↓

Title and abstract relevance assessment

↓

Full-text assessment where potentially relevant

↓

Application of inclusion and exclusion criteria

↓

43 substantive sources retained for integrative synthesis

↓

Foundational, methodological, statutory and standards-based sources recorded separately

The principal recorded exclusion categories were: insufficient relevance to employment-related relational access or governance; hybrid or remote work mentioned only incidentally; relational or governance processes peripheral to the analysis; insufficient substantive contribution to the theoretical problem; and a level of analysis that could not inform the proposed framework.
